# Heme Oxygenase-1 Suppresses Bovine Viral Diarrhoea Virus Replication *in vitro*

**DOI:** 10.1038/srep15575

**Published:** 2015-10-29

**Authors:** Chong Zhang, Fengxing Pu, Angke Zhang, Lele Xu, Na Li, Yunhuan Yan, Jiming Gao, Hongliang Liu, Gaiping Zhang, Ian G. Goodfellow, En-Min Zhou, Shuqi Xiao

**Affiliations:** 1 College of Veterinary Medicine, Northwest A&F University, No. 22 Xinong Road, Yangling, Shaanxi, 712100, China; 2 Experimental Station of Veterinary Pharmacology and Veterinary Biotechnology, Ministry of Agriculture, China, No. 22 Xinong Road, Yangling, Shaanxi, 712100, China; 3 College of Animal Science and Veterinary Medicine, Henan Agricultural University, Zhengzhou, Henan, 450002, China; 4 Division of Virology, Department of Pathology, University of Cambridge, Hills Road, Cambridge, CB2 2QQ, United Kingdom

## Abstract

Viral cycle progression depends upon host-cell processes in infected cells, and this is true for bovine viral diarrhoea virus (BVDV), the causative agent of BVD that is a worldwide threat to the bovine industry. Heme oxygenase-1 (HO-1) is a ubiquitously expressed inducible isoform of the first and rate-limiting enzyme for heme degradation. Recent studies have demonstrated that HO-1 has significant antiviral properties, inhibiting the replication of viruses such as ebola virus, human immunodeficiency virus, hepatitis C virus, and porcine reproductive and respiratory syndrome virus. However, the function of HO-1 in BVDV infection is unclear. In the present study, the relationship between HO-1 and BVDV was investigated. *In vitro* analysis of HO-1 expression in BVDV-infected MDBK cells demonstrated that a decrease in HO-1 as BVDV replication increased. Increasing HO-1 expression through adenoviral-mediated overexpression or induction with cobalt protoporphyrin (CoPP, a potent HO-1 inducer), pre- and postinfection, effectively inhibited BVDV replication. In contrast, HO-1 siRNA knockdown in BVDV-infected cells increased BVDV replication. Therefore, the data were consistent with HO-1 acting as an anti-viral factor and these findings suggested that induction of HO-1 may be a useful prevention and treatment strategy against BVDV infection.

Bovine viral diarrhoea (BVD) is one of the most economically important viral diseases affecting the cattle industry worldwide^1^,^2^. The etiologic agent of BVD is BVD virus (BVDV), which is a positive-strand RNA virus and a member of the genus *Pestivirus* in the family *Flaviviridae.* The family *Flaviviridae* includes three genera, the classical flaviviruses, the pestiviruses, and the hepatitis C viruses. Pestiviruses, comprising BVDV, border disease virus, and classical swine fever virus (CSFV), are important animal pathogens^2^. BVDV infections resulted in from $10 to $40 million losses per million calves in the United States alone^3^,^4^. In addition, it has been reported that BVDV were highly prevalent in Chinese pig herds^5^. Therefore, it is imperative to study BVDV biology for the development of antiviral strategies to combat BVDV infection.

Heme oxygenase-1 (HO-1) is a ubiquitously expressed inducible isoform of the first and rate-limiting enzyme of heme degradation and catabolizes free heme into biliverdin, carbon monoxide and iron^6^. HO-1 and its end-products have numerous biological functions, including anti-oxidant, anti-inflammatory, anti-apoptotic and anti-proliferative properties^7^. Recent studies have also demonstrated that HO-1 has significant antiviral properties^8^,^9^. Upregulation of HO-1 inhibits the replication of ebola virus (EBOV), hepatitis C virus (HCV), human immunodeficiency virus (HIV-1), and hepatitis B virus (HBV)^10^,^11^,^12^,^13^. Our previous work showed that induction of HO-1 expression inhibits significantly porcine reproductive and respiratory syndrome virus (PRRSV) replication^14^,^15^. However, the effect of HO-1 induction on BVDV infection is unknown.

In this study, the relationship between HO-1 and BVDV was investigated. The results showed that the expression of HO-1 was down-regulated in MDBK cells following BVDV infection, and that adenoviral-mediated overexpression or induction of HO-1 using the potent HO-1 inducer cobalt protoporphyrin (CoPP) effectively inhibited BVDV replication, which was reversed by prior siRNA knockdown. These results suggested that induction of HO-1 may be a useful prevention and treatment strategy against BVDV infection.

## Results

### BVDV infection decreases the expression level of HO-1 in MDBK cells

**Figure 1:**
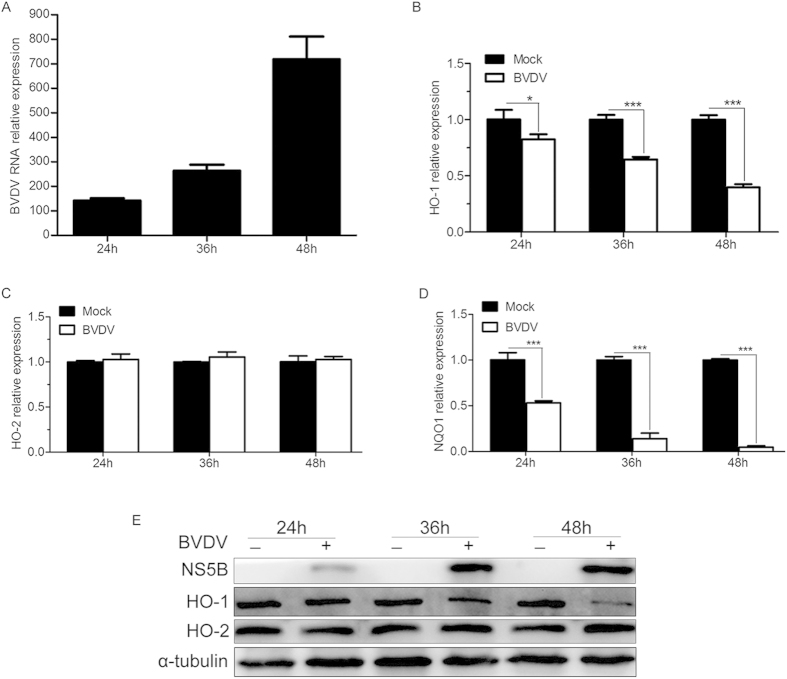
Effect of BVDV infection on HO-1 abundance. MDBK cells were infected with BVDV at an MOI of 0.1. The BVDV (**A**), HO-1 (**B**), HO-2 (**C**), NQO1 (**D**) mRNA and protein (**E**) expression levels after 24, 36 and 48 h of infection were analysed by qRT-PCR and Western blot, respectively. Values for cells uninfected with BVDV were set equal to 1. α-tubulin was used as loading control. The data represent the mean ± s.d. from three independent experiments, *P < 0.05, and ***P < 0.001.

To determine whether BVDV infection affects the expression of HO-1, we examined the abundance of BVDV, two antioxidant response element (ARE)-driven genes, including HO-1 and NADPH quinone oxidoreductase 1 (NQO1), and HO-2 (a constitutive heme oxygenase isoform but not regulated through the ARE) mRNA and protein in MDBK cells infected with or without BVDV at different times by qRT-PCR and Western blot. We observed that BVDV infection markedly reduced HO-1 expression. The reduction of HO-1 expression was progressive and time dependent (Fig. 1B,E), which showed a marked drop within 24 to 48 hours after virus infection as replication reached its peak (Fig. 1A,E). There was a 18%, 36%, and 60% decrease in the abundance of HO-1 mRNA at 24hpi, 36 hpi, and 48hpi, respectively (Fig. 1B). In the meantime there was a marked decrease of HO-1 protein abundance in MDBK cells infected with BVDV (Fig. 1E). In addition, BVDV infection also greatly reduced the expression level of NQO1 (Fig. 1D), which suggests that the decrease of HO-1 expression in BVDV infected cells might be a consequence of generalized inhibition of ARE-driven gene expression. But we did not observed a significant change in expression of HO-2 (Fig. 1C,E), suggesting that the reduction of HO-1 is relatively specific.

### CoPP attenuates BVDV replication *in vitro*

**Figure 2:**
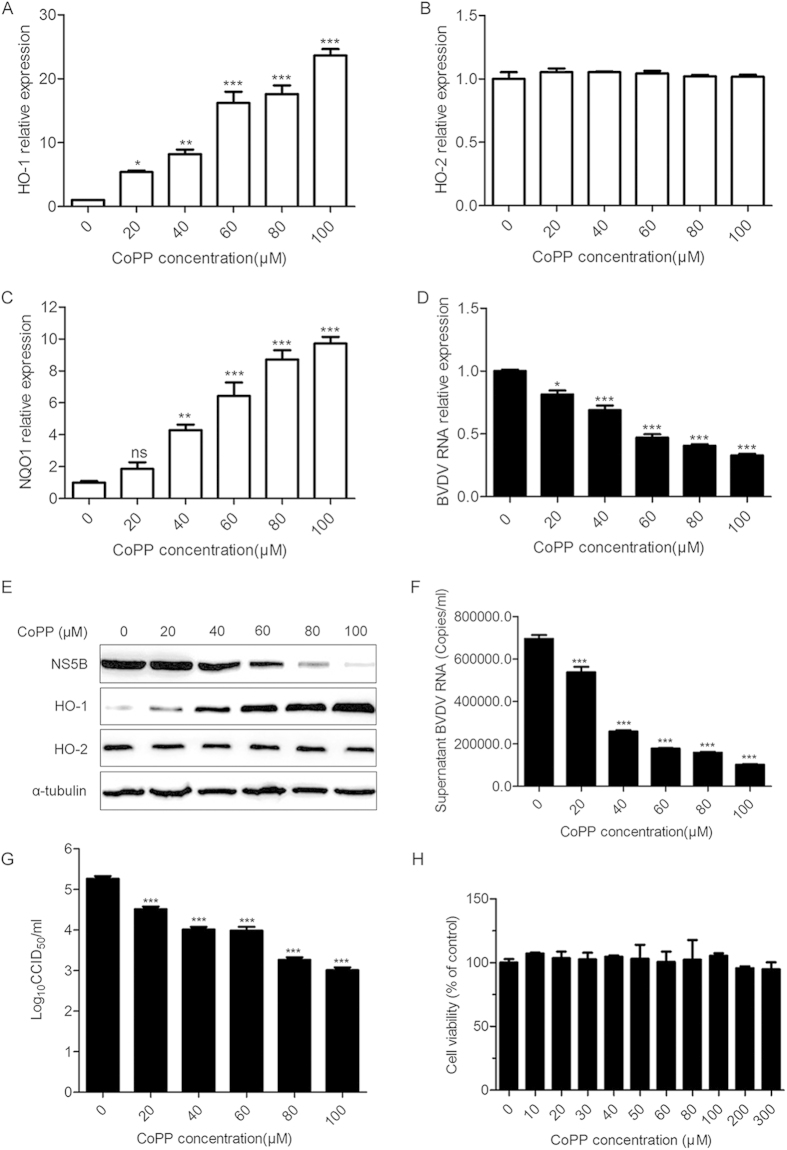
CoPP treatment attenuates BVDV replication in a dose-dependent manner. MDBK cells infected with BVDV were treated with various concentrations of CoPP for 36 h from 2 hpi, and then intracellular HO-1 (**A**), HO-2 (**B**), NQO1 (**C**), BVDV RNA (**D**) and protein (**E**), extracellular BVDV RNA (**F**) and virus titers (**G**) in the supernatants were analyzed by qRT-PCR, Western blot, and CCID_50_. (**H**) Cell toxicity was examined in MDBK cells using CCK-8 and was expressed as relative cell viability by comparing with the viable cells in the absence of CoPP (set up as 100%). Data are expressed as mean ± s.d. of three independent experiments, *P < 0.05, **P < 0.01, and ***P < 0.001.

BVDV infection significantly decreased the abundance of HO-1 in MDBK cells, indicative of potential function in BVDV biology. To investigate this, MDBK cells were incubated with the indicated concentrations of CoPP (a classical inducer of HO-1) 2 h after viral exposure, and then the expression of HO-1, HO-2 and NQO1, viral RNA and protein expression, and BVDV production were analyzed by qRT-PCR, Western blot, virus titers at 36 hpi. As shown in Fig. 2, CoPP substantially induced HO-1 (Fig. 2A), NQO1 (Fig. 2C) but not HO-2 (Fig. 2B) expression in MDBK cells in a dose-dependent manner, as predicted from the mode of action of CoPP as an inducer of HO-1. Treatment of MDBK cells post-infection with CoPP markedly reduced intracellular BVDV RNA (Fig. 2D), and intracellular BVDV protein (Fig. 2E), the levels of BVDV RNA in the supernatants (Fig. 2F) and virus titers (Fig. 2G). The dose-dependent increase (5-fold to 24-fold) in the abundance of HO-1 (Fig. 2A) was parallel with a 19% to 67% reduction in the amount of viral mRNA (Fig. 2D), viral protein (Fig. 2E), and a 0.75 log10 to 2.25 log10 reduction in the BVDV progeny titers (Fig. 2G). And the amount of extracellular viral RNA in CoPP-pretreated MDBK cells decreased by a 23% to 85% compared with untreated controls (Fig. 2F). To ensure whether CoPP treatment was cytotoxic to MDBK cells, cell viability assays were performed. The results showed that there were no significant cytotoxic effects at the CoPP concentrations used (Fig. 2H).

**Figure 3:**
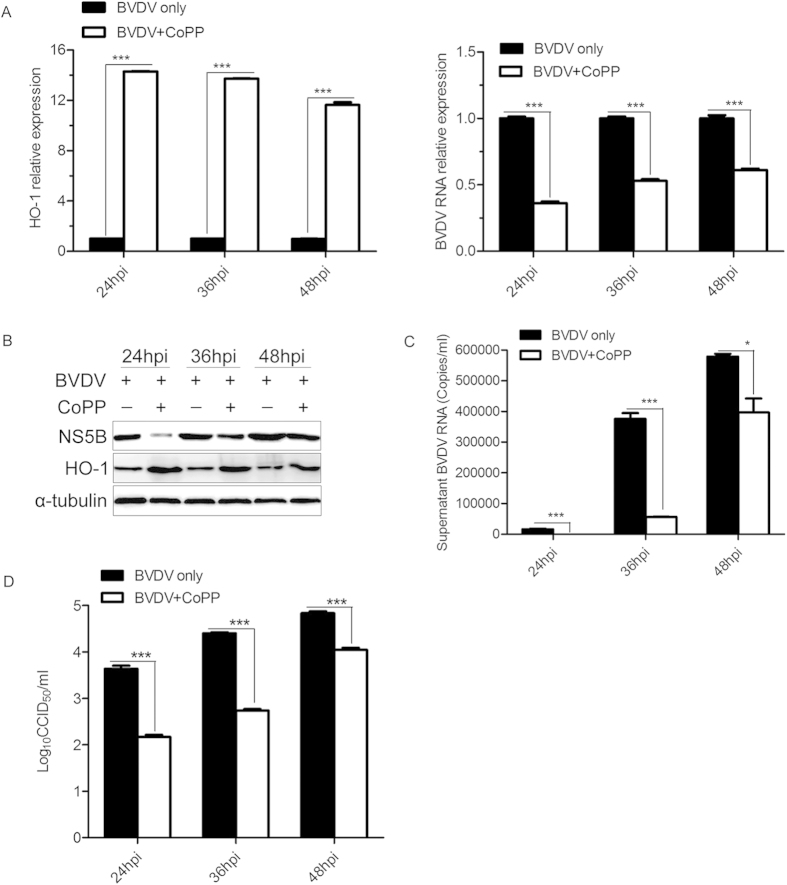
CoPP pretreatment inhibits BVDV replication in MDBK cells. MDBK cells were treated with CoPP (80 μM) 12 h, before BVDV (MOI of 0.1) infection, and then cells and culture supernatants were collected at the indicated times to detect intracellular HO-1, BVDV RNA (**A**) and protein (**B**), extracellular BVDV RNA (**C**) and virus titers (**D**) in the supernatants. Data are expressed as mean ± s.d. of three independent experiments, *P < 0.05, and ***P < 0.001.

To further confirm the effect of CoPP treatment on BVDV replication, MDBK cells were pretreated with CoPP for 12 h before BVDV infection, supernatants and cells were harvested at the indicated times, and then virus titers and viral gene expression were examined. As expected, pretreatment with CoPP in MDBK cells considerably decreased the expression level of intracellular BVDV RNA (Fig. 3A) and protein (Fig. 3B), also reduced the levels of BVDV RNA (Fig. 3C) and virus titers (Fig. 3D) in the supernatants, compared with negative control. Expression levels of intracellular viral RNA in CoPP-pretreated MDBK cells were decreased by ~39 to 64% (Fig. 3A). Virus titers in the supernatants decreased by ~0.79 to 1.66 log10 (Fig. 3D), and expression level of extracellular viral RNA in CoPP-pretreated MDBK cells were down-regulated by ~31 to 97% (Fig. 3C).

**Figure 4:**
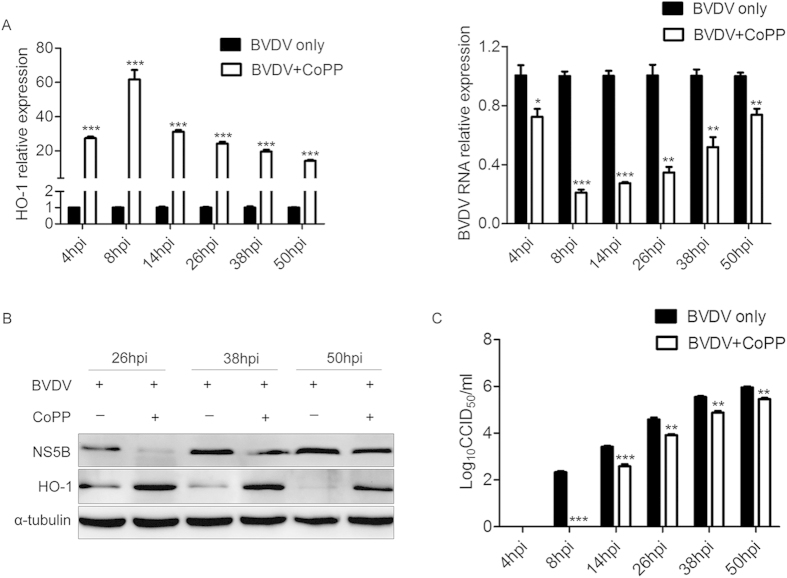
CoPP treatment suppresses BVDV of post-infection viral replication. MDBK cells infected with BVDV at a MOI of 1 were treated with 80 μM CoPP at the indicated times from 2 hpi, and then intracellular HO-1, BVDV RNA (**A**) and protein (**B**), and virus titers (**C**) in the supernatants were analyzed by qRT-PCR, Western blot, and CCID_50_. Data are expressed as mean ± s.d. of three independent experiments, *P < 0.05, **P < 0.01, and ***P < 0.001.

To determine the ability of CoPP-induced HO-1 to suppress post-entry viral replication, MDBK cells were incubated with 80 μM CoPP 2 h after 1 MOI of viral exposure, and then HO-1, viral gene expression and virus titers were analyzed at the indicated times (Fig. 4). As shown in Fig. 4A left, HO-1 was significantly upregulated by CoPP in BVDV infected cells; and it reached its peak expression at 8hpi, and then its expression decreased gradually as the infection progressed but still remained markedly higher than the level in the BVDV infection control. At the same time, the levels of intracellular BVDV RNA (Fig. 4A right), viral protein (Fig. 4B) and virus titers in the supernatants (Fig. 4B) were reduced significantly. Expression levels of intracellular viral RNA in CoPP-treated MDBK cells were decreased by ~28 to 89% (Fig. 4A right). Virus titers in the supernatants decreased by ~0.5 to 2.33 log10 (Fig. 4C). These results demonstrate that CoPP upregulates HO-1 expression and concomitantly inhibits BVDV replication without affecting cell viability.

### Overexpression of HO-1 inhibits BVDV replication

**Figure 5:**
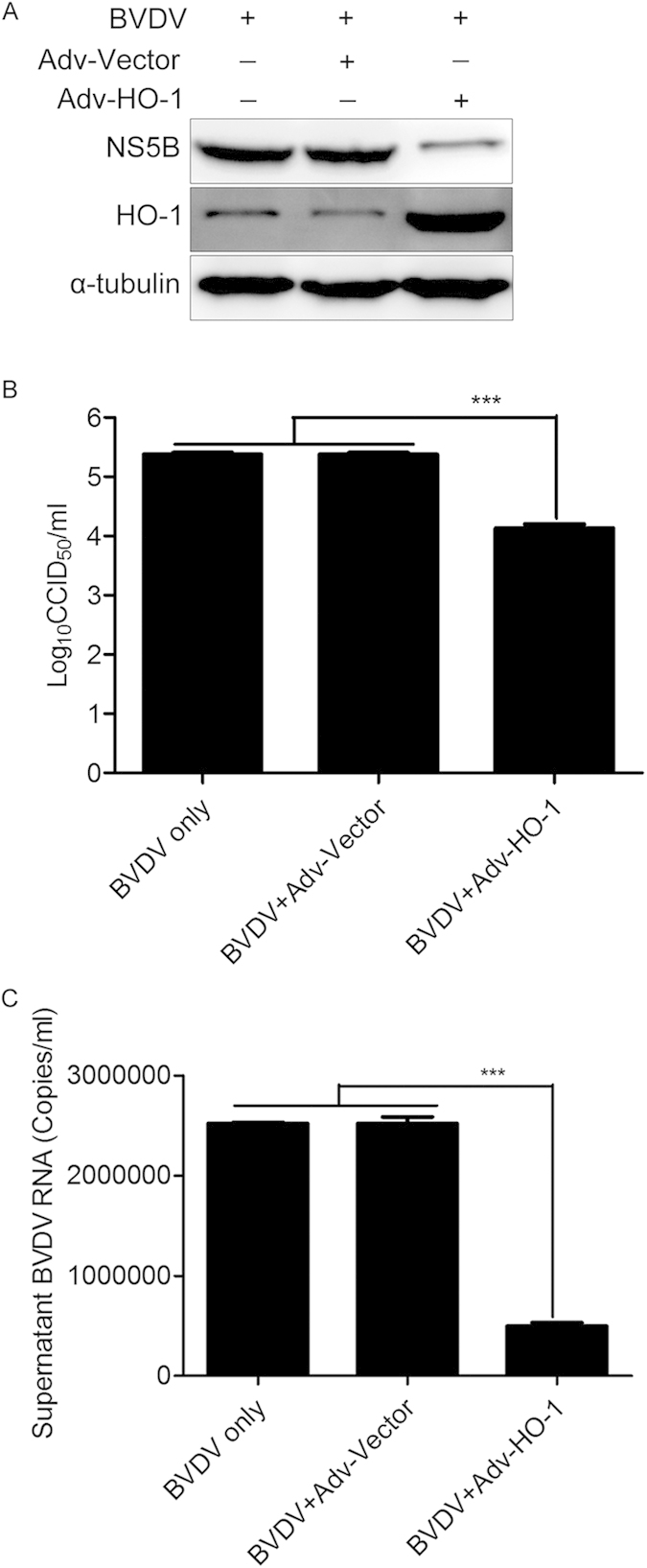
Overexpression of HO-1 inhibits BVDV replication. MDBK cells were infected with either adenovirus vector (Adv-Vector) or Adv-HO-1 at a MOI of 5.0 for 24 h, followed by BVDV (MOI of 0.1) infection for 36 h. Then intracellular HO-1 and BVDV NS5B protein expression (**A**), virus titers (**B**) and BVDV RNA (**C**) in the supernatants were analyzed by Western blot, CCID_50_, and qRT-PCR. Data are expressed as mean ± s.d. of three independent experiments, ***P < 0.001.

To further determine that HO-1 is a mediator of CoPP-dependent inhibition of BVDV replication, we examined whether direct HO-1 expression could inhibit BVDV replication. A recombinant adenovirus (Adv) that over expressed HO-1 was used to increase the abundance of HO-1. MDBK cells were infected with either Adv-Vector (as a control) or Adv-HO-1 for 24 h, and then infected with BVDV for 36 h. As shown in Fig. 5A, the expression levels of BVDV NS5B protein from the cells co-infected with the Adv-HO-1 and BVDV were markedly decreased in comparison with the cells co-infected with Adv-Vector control and BVDV. Adenovirus-mediated HO-1 overexpression significantly decreased virus titers in the supernatants by 1.25log10 (Fig. 5B), and expression level of extracellular viral RNA by 80% (Fig. 5C). These results indicate that HO-1 can directly inhibit BVDV replication.

### Decreased basal levels of HO-1 promotes BVDV replication

**Figure 6:**
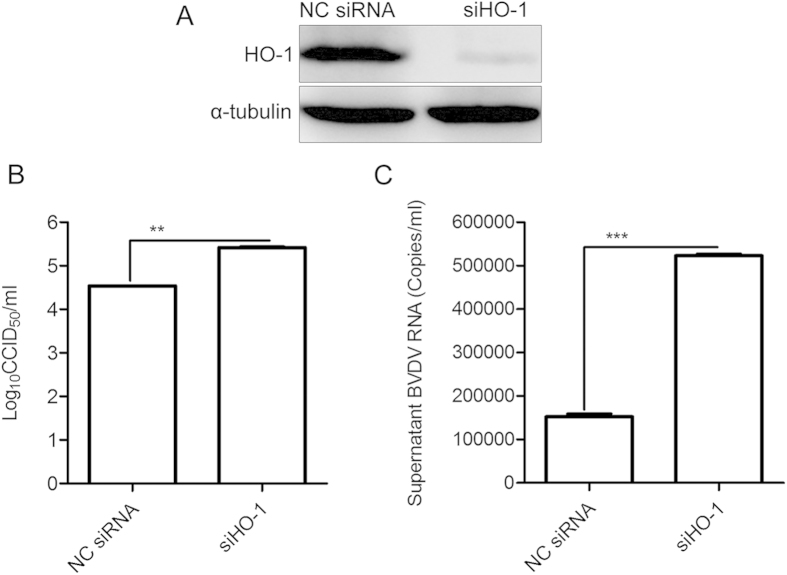
HO-1 knockdown promotes BVDV replication. MDBK cells were transfected with either non-targeting control (NC) siRNA or siRNA targeting HO-1, for 12 h followed by infection with BVDV at a MOI of 0.1 for 36 h. Reduced HO-1 expression was verified by Western blot analyze using antibody against HO-1 (**A**). Virus titers (**B**) and BVDV RNA (**C**) in the supernatants were analyzed by CCID_50_ and qRT-PCR. Data are expressed as mean ± s.d. of three independent experiments, **P < 0.01, and ***P < 0.001.

Because induction of HO-1 by CoPP inhibited BVDV replication, it is important to define whether decreased basal levels of HO-1 have an impact on BVDV replication. RNA interference was used to deplete HO-1 in MDBK cells, and the effect on BVDV replication was examined. As shown in Fig. 6A, the expression of HO-1 protein was considerably reduced after transfection of siRNA against HO-1. Subsequently, infection with BVDV showed an HO-1 siRNA-mediated increase of virus replication. Virus titers in the supernatants increased by 0.88log10 (Fig. 6B), and BVDV RNA levels up-regulated by 240% (Fig. 6C) in cells transfected with siHO-1.

### CoPP-mediated attenuation of BVDV replication is HO-1 dependent

**Figure 7:**
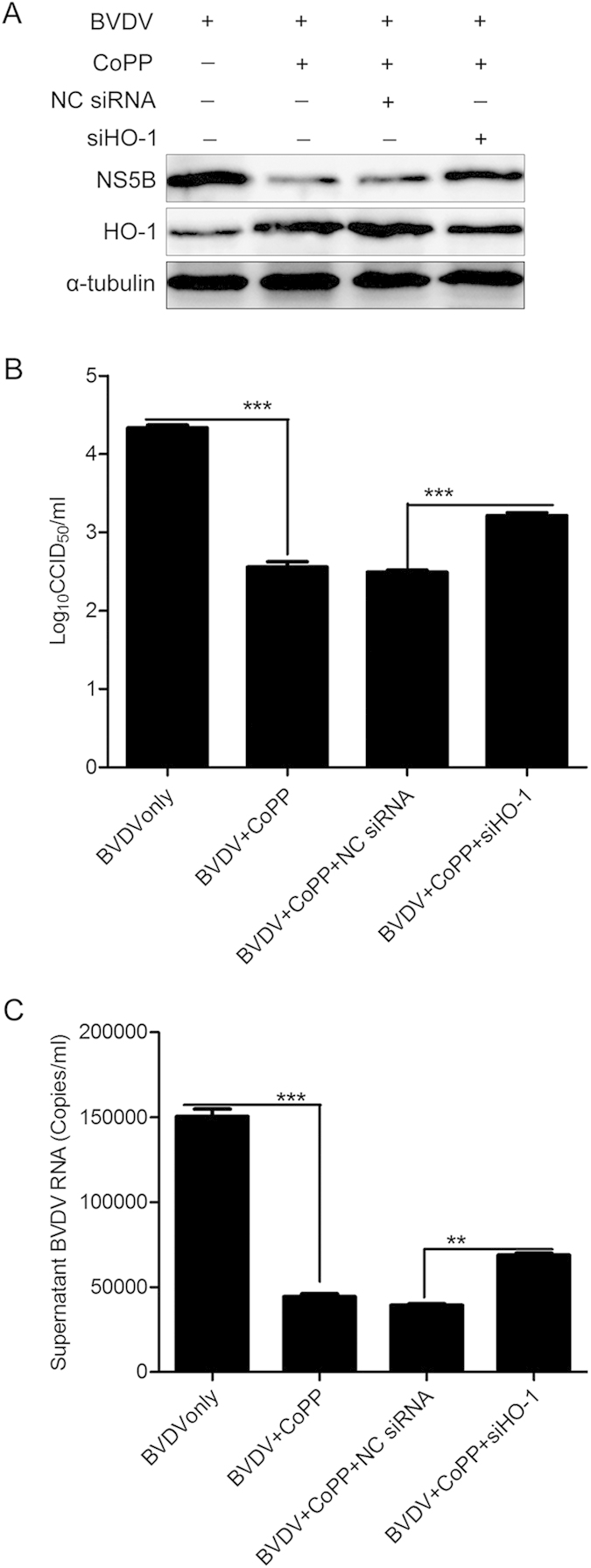
CoPP-induced attenuation of BVDV is HO-1 dependent. siRNAs specific to HO-1 were transfected into MDBK cells, which were then treated with CoPP for 12 h. And then, these cells were infected with BVDV at a MOI of 0.1 for 36 h. Subsequently, the intracellular HO-1 and BVDV NS5B protein expression levels were analyzed by Western blot (**A**), and α-tubulin was used as the control. Virus titers (**B**) and BVDV RNA (**C**) in the supernatants were analyzed by CCID_50_ and qRT-PCR. Data are expressed as mean ± s.d. of three independent experiments, **P < 0.01, ***P < 0.001.

To further confirm that inhibition of BVDV replication by CoPP is dependent on HO-1 induction, MDBK cells were transfected with siRNAs against HO-1 for 12 h, and then treated with CoPP and infected with BVDV at an MOI of 0.1 for 36 h. Pretreatment with HO-1 siRNA partially reversed the inhibitory effect of HO-1 induction by CoPP on BVDV replication compared with NC siRNA treatment, substantially increasing the abundance of intracellular BVDV NS5B protein (Fig. 7A), virus titers in the supernatants by 0.72log10 (Fig. 7B). Expression levels of extracellular BVDV RNA in HO-1 siRNA-pretreated MDBK cells were increased by 75% (Fig. 7C). These results indicated that CoPP-mediated inhibition of BVDV replication is partially dependent on HO-1 up-regulation.

## Discussion

HO-1 is rapidly induced in response to various stimuli such as oxidants, heat shock, and infection with microbial agents, including viruses. For virus infection, human cytomegalovirus (HCMV) and classical swine fever virus (CSFV) induced HO-1 expression^16^,^17^, and over expression of the HCV core-NS3 protein increased also HO-1 expression^18^. Consequently, it might be anticipated that HO-1 would be up-regulated in response to BVDV infection. In contrast, our findings revealed that BVDV infection markedly decreases the expression of HO-1 in MDBK cells (Fig. 1). Similar to our observations, spring viremia of carp virus (SVCV)^19^, enterovirus 71 (EV71)^20^, porcine reproductive and respiratory syndrome virus (PRRSV)^21^,^22^, HIV^23^, and HCV^24^,^25^ infecton down-regulated also HO-1 expression. These studies indicated the ability of virus to modulate levels of HO-1 *in vitro* and *in vivo*, and different viruses produced different HO-1 regulation.

Although our data demonstrated that BVDV infection modulates expression of HO-1 in MDBK cells, it is not clear whether HO-1 plays an anti- or pro-viral role in BVDV infection. Previous studies have shown that induction or over-expression of HO-1 can inhibit virus replication and ameliorate the viral infection^10^,^11^,^12^,^26^,^27^. Upregulation of HO-1 by CoPP treatment, pre- and postinfection, remarkably inhibits ebola virus (EBOV) transcription/replication^13^. Induction or overexpression of HO-1 also suppresses hepatitis C virus (HCV) replication and protects hepatocytes from oxidative damage^10^. Our results clearly showed that HO-1 induction by CoPP significantly suppressed BVDV replication (Figs 2-4). Although there was a substantially lower BVDV infectivity relative to the untreated controls, detectable BVDV RNA and NS5B protein in the infected cells pretreated with CoPP suggested that viral entry is substantially inhibited, but not completely blocked (Fig. 3). Treatment of BVDV-infected cells with CoPP also significantly suppressed BVDV replication (Fig. 4). Therefore, decreased viral entry into MDBK cells may not be the only factor responsible for the inhibition of BVDV infection. Despite the presence of viral RNA and protein in BVDV-infected cells, the inability of BVDV to largely replicate strongly suggested a putative role of CoPP-induced HO-1 in suppressing the virus post entry events.

To determine the specificity of HO-1 induction by CoPP-mediated attenuation of BVDV replication, recombinant adenovirus was used to overexpression HO-1 (Fig. 5) and RNA interference was used to knockdown HO-1. HO-1 knockdown by specific siRNA reduced HO-1 expression and concomitantly increased the level of BVDV RNA and progeny virus production (Fig. 6), suggesting that decreased basal levels of HO-1 have the impact on BVDV replication. Further, the fact that HO-1 knockdown by siRNA reversed the inhibitory effect of HO-1 induction by CoPP on BVDV replication suggests a direct correlation between HO-1 expression and the inhibition of BVDV replication. The mechanism by which HO-1 induction impacts BVDV replication will require further analysis, in the case of Ebola virus, the mechanism of HO-1 mediated inhibition of replication also remains to be fully determined, but it is known to affect a post entry event, most likely transcription and/or replication^13^. HO-1 induction leads to increased heme degradation and the conversion of free heme into biliverdin, carbon monoxide and iron^6^. These byproducts may be responsible for the observed inhibition of BVDV replication as previous studies with HCV have demonstrated that the production of increased levels of biliverdin lead to the induction of the innate immune response^28^. Such an effect would result in the establishment of an antiviral state in permissive cells, leading to reduced viral replication via a number of inhibitory mechanisms. Further studies to elucidate the precise step of the BVDV life cycle affected by HO-1 and the mechanism by which this step is inhibited are currently underway and will form the focus of future studies.

In conclusion, the present study demonstrated that BVDV infection decreases the expression level of HO-1, and induction of HO-1 by CoPP attenuates BVDV infection *in vitro*. The CoPP-mediated inhibition of BVDV replication is dependent upon HO-1 expression. These results suggested that HO-1 induction is a useful prevention and treatment strategy against BVDV infection and warrant further *in vivo* evaluation.

## Methods

### Cells, viruses and chemicals

Madin-darby Bovine Kidney (MDBK) cells were maintained in Dulbecco’s modified Eagle’s medium (DMEM; Life Technologies Corporation, New York, CA, USA) supplemented with 10% fetal bovine serum (FBS) and 1% antibiotic-antimycotic (Life Technologies Corporation, New York, CA, USA) at 37 °C and 5% CO_2_. BVDV strain Oregon C24V (CVCC no. AV69) was propagated on MDBK cells and stored at –70°C. Protoporphyrin IX cobalt chloride (CoPP), a classical inducer of HO-1 gene expression, was purchased from Sigma-Aldrich (St. Louis, MO, USA).

### Cell viability assay

The cytotoxicity of CoPP was evaluated by the Cell Counting Kit-8 (CCK-8) assay (Beyotime, Nanjing, China). MDBK cells were added to each well of 96-well plates (1 × 10^4^/well). After culturing for 24 h at 37 °C with 5% CO_2_, CoPP was added at specific concentration and cultured for 48 h. Then the 10 μL CCK-8 reagent was added to each well of a 96-well plate containing 100 uL culture medium according to the manufacturer’s instructions and incubated for 2 h at 37 °C. Viable cells were evaluated by absorbance measurements at 450 nm. Results were expressed as relative to the optical density of wells containing untreated control cells defined as 100% viability.

### Quantitative reverse transcriptase-PCR (qRT-PCR)

Total RNA from MDBK cells, or supernatant BVDV RNA was isolated using the TRIzol reagent and reverse transcribed using Primescript RT reagent Kit (TaKaRa, Dalian, China) according to the manufacturer’s instructions. Quantitative PCR (qPCR) reaction was performed on Step One Plus® real-time PCR system (Applied Biosystems, Foster City, CA, USA) using FastStart Universal SYBR green master (Roche, Basle, Switzerland), forward and reverse primers for the HO-1, HO-2, NQO1 and the RNA of BVDV. Beta-2-microglobulin (B2M) mRNA was used as an internal reference.

For detection of supernatant BVDV RNA, a plasmid bearing a 357bp fragment of the BVDV 5’UTR sequence was used to generate a standard curve. The standard curve was plotted from the results of parallel PCR reaction performed on serial dilutions of standard DNA. RNA absolute quantities were calculated by normalization to the standard curve. The primers used for qPCR amplification are listed in Table 1.

### Western blot analysis

Western blotting was processed as described previously^14^. Briefly, MDBK cells were lysed and the cellular proteins were separated by SDS-PAGE and transferred onto a polyvinylidene difluoride (PVDF) membrane. The PVDF membrane was probed with one of the following primary antibodies, mouse anti-HO-1 polyclonal antibodies at a 1:1000 dilution and mouse anti-NS5B of BVDV polyclonal antibodies at a 1:1000 dilution produced in our laboratory, anti-HO-2 antibody at a 1: 1000 (Abcam, catalog#: ab90492, Cambridge, UK), anti-α-tubulin antibody at a 1:5000 (Abcam, catalog#: ab7750, Cambridge, UK), and then with HRP-conjugated anti-mouse IgG at a 1:2000 (Jackson, catalog#: 111-035-003, West Grove, PA, USA) as the secondary antibody. Immunolabeled proteins were visualized using ECL reagent (Pierce, Rockford IL, CA, and USA).

### Virus titration

Virus progeny production was determined by titration as described previously^29^. Briefly, MDBK cells were trypsinized and seeded in 96-well plate 24 hours before virus infection. Virus supernatants were prepared through serial dilutions and 100 μl of solution was added to each well in replicates of eight. Six days after infection, the 50% cell culture infection dose (CCID50) was calculated by the Reed-Muench method.

### Overexpression of HO-1

Recombinant adenovirus-mediated HO-1 overexpression was performed as described previously^14^. Briefly, MDBK cells were infected with either Adv-Vector (as a control) or Adv-HO-1 for 24 h, and then infected with BVDV at a MOI of 0.1. Cells and supernatant were harvested at 36 hours post-infection (hpi) of BVDV for assaying the expression of HO-1 and viral protein, viral genome and progeny virus production.

### siRNA knockdown of HO-1

MDBK cells were transfected with specific siRNA targeting HO-1 or a non-targeting siRNA control (100 nM final concentration) according to the manufacturer’s protocol (RIBOBIO, Guangzhou, China), followed by infection with BVDV at a MOI of 0.1. Viral genome replication and progeny virus production were analyzed at 36 hpi by qRT-PCR and CCID_50_.

### Statistical analysis

All experiments were performed with at least three independent experiments, and representative data from all experiments are shown or expressed as the mean ± s.d. of the independent experiments. Statistical significance was determined by Two-tailed Student’s *t test* when only two groups were compared or by one-way analysis of variance (ANOVA) accompanied with Turkey multiple-comparisons test (GraphPad Prism version 5.0) when more than two groups were compared. A *P* value < 0.05 was considered statistically significant.

## Additional Information

**How to cite this article**: Zhang, C. *et al.* Heme Oxygenase-1 Suppresses Bovine Viral Diarrhoea Virus Replication *in vitro*. *Sci. Rep.* **5**, 15575; doi: 10.1038/srep15575 (2015).

## Figures and Tables

**Table 1:** List of primers for real-time PCR.

Gene	Primer	Sequence (5′-3′)
Bovine HO-1	Forward primer	CAGAAGATGTAGCCAGAGCA
	Reverse primer	CATAGGGCAAGCGGTCA
Bovine HO-2	Forward primer	TGAGTTCAACATGCAGGTGTTC
	Reverse primer	CAGCGTAGTAGGGGCACTTC
Bovine NQO1	Forward primer	GGCTGTCAGAAAAGCACTGATC
	Reverse primer	ACAGTCTCGGCAGGATACTGAA
Bovine B2M	Forward primer	AGCAAGGATCAGTACAGCTGCCG
	Reverse primer	ATGTTCAAATCTCGATGGTGCTGCT
BVDV 5’UTR1	Forward primer	CAGTGGTGAGTTCGTTGGATGGCT
	Reverse primer	AGCACCCTATCAGGCTGTATTCGTAAC
BVDV 5’UTR2	Forward primer	GCGAAGGCCGAAAAGAGGCTAG
	Reverse primer	CCGACGGGTTTTTGTTTGTATGTT

Note: The primers from BVDV 5′UTR2 were used to construct plasmid for generating a standard curve.
